# Quantitative Proteomics Reveals a Role for SERINE/ARGININE-Rich 45 in Regulating RNA Metabolism and Modulating Transcriptional Suppression *via* the ASAP Complex in *Arabidopsis thaliana*

**DOI:** 10.3389/fpls.2019.01116

**Published:** 2019-09-19

**Authors:** Samuel L. Chen, Timothy J. Rooney, Anna R. Hu, Hunter S. Beard, Wesley M. Garrett, Leann M. Mangalath, Jordan J. Powers, Bret Cooper, Xiao-Ning Zhang

**Affiliations:** ^1^Bioinformatics Program, St. Bonaventure University, St. Bonaventure, NY, United States; ^2^Biochemistry Program, St. Bonaventure University, St. Bonaventure, NY, United States; ^3^Soybean Genomics and Improvement Laboratory, USDA-ARS, Beltsville, MD, United States; ^4^Animal Biosciences & Biotechnology Laboratory, USDA-ARS, Beltsville, MD, United States; ^5^Department of Biology, St. Bonaventure University, St. Bonaventure, NY, United States

**Keywords:** ACINUS, apoptosis and splicing-associated protein complex, *Arabidopsis thaliana*, inflorescence, quantitative proteomics, RNA metabolism, Sin3-associated protein 18, SERINE/ARGININE-rich 45

## Abstract

Pre-mRNA alternative splicing is a conserved mechanism for eukaryotic cells to leverage existing genetic resources to create a diverse pool of protein products. It is regulated in coordination with other events in RNA metabolism such as transcription, polyadenylation, RNA transport, and nonsense-mediated decay *via* protein networks. SERINE/ARGININE-RICH 45 (SR45) is thought to be a neutral splicing regulator. It is orthologous to a component of the apoptosis and splicing-associated protein (ASAP) complex functioning to regulate RNA metabolism at multiple levels. Within this context, we try to understand why the *sr45-1* mutant Arabidopsis has malformed flowers, delayed flowering time, and increased disease resistance. Prior studies revealed increased expression for some disease resistance genes and the flowering suppressor *Flowering Locus C* (*FLC*) in *sr45-1* mutants and a physical association between SR45 and reproductive process-related RNAs. Here, we used Tandem Mass Tag-based quantitative mass spectrometry to compare the protein abundance from inflorescence between Arabidopsis wild-type (Col-0) and *sr45-1* mutant plants. A total of 7,206 proteins were quantified, of which 227 proteins exhibited significantly different accumulation. Only a small percentage of these proteins overlapped with the dataset of RNAs with altered expression. The proteomics results revealed that the *sr45-1* mutant had increased amounts of enzymes for glucosinolate biosynthesis which are important for disease resistance. Furthermore, the mutant inflorescence had a drastically reduced amount of the Sin3-associated protein 18 (SAP18), a second ASAP complex component, despite no significant reduction in *SAP18* RNA. The third ASAP component protein, ACINUS, also had lower abundance without significant RNA changes in the *sr45-1* mutant. To test the effect of SR45 on SAP18, a SAP18-GFP fusion protein was overproduced in transgenic Arabidopsis Col-0 and *sr45-1* plants. SAP18-GFP has less accumulation in the nucleus, the site of activity for the ASAP complex, without SR45. Furthermore, transgenic *sr45-1* mutants overproducing SAP18-GFP expressed even more *FLC* and had a more severe flowering delay than non-transgenic *sr45-1* mutants. These results suggest that SR45 is required to maintain the wild-type level of SAP18 protein accumulation in the nucleus and that *FLC*-regulated flowering time is regulated by the correct expression and localization of the ASAP complex.

## Introduction

In eukaryotic cells, pre-mRNA alternative splicing is a conserved mechanism to increase the diversity of mature transcripts and their protein products. The spliced mature mRNAs with proper 5’-7-methylguanosine capping and 3’-polyadenylation are resistant to immediate degradation by cellular machinery and are viable templates for translation. A successful splicing event consists of several sequential steps: splicing factors recruiting spliceosome components; spliceosome components aggregating in sequence to recognize splice sites; catalysis involving the 5’ splice site, branch site, and 3’ splice site; the release of the excised intron and spliced mRNA; and a conclusion with spliceosome disassembling. This process is energy-dependent and is regulated in coordination with other events in RNA metabolism such as transcription, polyadenylation, nuclear export, and nonsense-mediated decay (NMD). These related events are coordinated through a network of protein players, including the SR proteins, a family of known splicing regulators (Fu and Ares, 2014).

Evidence suggests that the SERINE/ARGININE-rich 45 (SR45) protein in *Arabidopsis thaliana* acts as a neutral splicing regulator that could trigger nearby splicing activation or suppression events (Zhang et al., 2017). SR45, an RNA-binding protein, is orthologous to RNPS1 in humans and other animals (Zhang and Mount, 2009). RNPS1 is a component of the apoptosis and splicing-associated protein (ASAP) complex (Schwerk et al., 2003), which functions to regulate RNA metabolism at multiple levels (Deka and Singh, 2017). The two other core proteins in the ASAP complex are SAP18 and ACINUS (Schwerk et al., 2003). A general understanding of the function of the ASAP complex has been mostly focused on transcriptional repression because SAP18 can bind to the mSIN3 transcriptional repressor to recruit histone deacetylases (HDACs) to induce transcriptional silencing in mammalian cells (Zhang et al., 1997).

In addition to functioning in the ASAP complex, RNPS1 is a peripheral component of the conserved RNA quality control machinery exon junction complex (EJC) and is involved in splicing regulation and communication with NMD (Lykke-Andersen et al., 2001), a surveillance process that removes mRNA transcripts harboring premature stop codons (PTCs). The multifaceted involvement of RNPS1 in transcriptional regulation, splicing, and RNA quality control denotes its significance in the regulatory network for RNA metabolism. Due to SR45’s orthology to RNPS1, it is likely that SR45 has these RNPS1 functions.

*A. thaliana* with the *sr45-1* null mutation exhibits both vegetative and reproductive defects such as smaller stature with narrower leaves and flower petals, delayed root growth, late flowering, and a mild sterility during seed formation (Ali et al., 2007; Zhang et al., 2017). The pre-mRNA of the *SR45* gene is alternatively spliced into two functional isoforms, *SR45.1* and *SR45.2*. The protein product of these alternative transcripts has distinct functions: SR45.1 is mostly involved in flower development and SR45.2 plays a bigger role in proper root growth (Zhang and Mount, 2009). The two isoforms differ by 7 amino acids which are missing in SR45.2. Within this alternative fragment, a phosphorylation event on threonine 218 is instrumental in bringing about the separate functions of the two transcripts (Zhang et al., 2014). Independent efforts have been put forth to understand the molecular mechanisms that SR45.1 employs during plant reproduction (Ali et al., 2007; Ausin et al., 2012; Zhang et al., 2014; Questa et al., 2016; Zhang et al., 2017). The current understanding suggests that in the inflorescence SR45 is associated with RNAs functioning in a wide range of processes, from splicing to reproduction, and that SR45-dependent alternative splicing events are overrepresented in transcripts for RNA binding proteins and RNA splicing (Zhang et al., 2017). Although the exact mechanisms have not been proven, it is possible that the fate of a splicing event is determined *via* direct SR45-RNA interaction or indirectly by association with other SR45-associated proteins, such as splicing factors and spliceosome components (Golovkin and Reddy, 1999; Day et al., 2012; Baldwin et al., 2013; Zhang et al., 2014; Stankovic et al., 2016).

There are, however, other *A. thaliana* phenotypes of the *sr45-1* mutant that are not as easily explained by altered RNA splicing events. For example, there is an elevated RNA level of *FLOWER LOCUS C* (*FLC*) in the *sr45-1* mutant (Ali et al., 2007). A recent study discovered the presence of the ASAP complex at the *FLC* locus, which suggests that the ASAP complex recruits HDACs to the *FLC* locus for transcriptional repression of *FLC* (Questa et al., 2016). This finding expanded the narrow focus of SR45 as a splicing regulator to a chromatin-level transcriptional control factor. In mammalian cells, it has been found that epigenetic changes could affect the rate of transcription and the subsequent outcome in RNA splicing (Warns et al., 2016). We have found that SR45 is physically associated with the *FLC* RNA, but *FLC* RNA was not alternatively spliced in the *sr45-1* mutant (Zhang et al., 2017). Questions still remain as to whether there is any coordination between SR45-regulated splicing events and chromatin modification for the same gene. Interestingly, *FLC* is not the only flowering-related gene that SR45 regulates. The *sr45-1* mutant also displays a lower frequency of DNA methylation at the *FLOWERING WAGENINGEN* (*FWA*) locus and a reduction of the RNA-dependent DNA methylation (RdDM) pathway, which is associated with the delayed flowering phenotype in the *sr45-1* mutant (Ausin et al., 2012). Thus, SR45 may regulate RdDM components that coordinate gene silencing for flowering.

In addition, the *sr45-1* mutant has enhanced resistance to *Pseudomonas syringae* pv. *maculicola* strain DG3 and *Hyaloperonospora parasitica* isolate Noco2 and has higher levels of callose deposition at the cell wall, reactive oxygen species, and salicylic acid (Zhang et al., 2017). SR45 also exhibits a strong preference in suppressing plant innate immunity genes such as *PR1, PR5, ACD6*, and *PAD4* (Zhang et al., 2017) which cannot be explained by alternative splicing alone. Consequently, SR45 is considered a suppressor of innate immunity in *A. thaliana.*

These observations urge an exploration of the possibility that SR45 is more than just a splicing regulator. To better understand the proteomic landscape in which SR45 acts to affect RNA metabolism in inflorescence tissue, we evaluated inflorescence proteins from wild-type *A. thaliana* (Col-0) and *sr45-1* mutants by quantitative tandem mass spectrometry. We identified 227 differentially accumulated proteins and predicted their roles in RNA metabolism and other biological processes. Our data shows that SR45 likely functions through the ASAP complex to suppress *FLC* and immunity genes.

## Materials and Methods

### Plant Growth Condition

All *A. thaliana* plants used in this study are in the *Colombia* (Col-0) background. The *sr45-1* (SALK_004132) mutant plant was originally obtained from the *Arabidopsis Biological Resource Center* (ABRC). Primers used to confirm T-DNA insertion in *sr45-1* were described (Zhang and Mount, 2009) and are listed in **Supplemental Table S1**. All plants were grown in soil (Sunshine #8, Griffin Greenhouse & Nursery Supplies), under a long-day (LD) condition of a 16/8 h photoperiod with light intensity of 100 μmol m^−2^ s^−1^ at 22 °C or otherwise specified.

### Total Protein Extraction

Healthy inflorescence tissues were harvested from 6-week old plants, and ground into fine powder using liquid nitrogen. Total protein was extracted according to the protocol from the Plant Total Protein Extraction Kit (PE0230, Sigma).

### Peptide Preparation

Protein concentration was determined by bicinchoninic assay (Pierce, Rockford, IL, USA). Proteins (∼300 µg), dissolved in urea, were reduced in 5 mM Tris(2-carboxyethyl)phosphine for 20 min, carboxyamidomethylated with 20 mM iodoacetamide for 20 min, and digested overnight at 37°C with Poroszyme immobilized trypsin (Thermo Fisher Scientific, Waltham, MA). The digested peptides were purified by reverse phase chromatography using SPEC-PLUS PT C18 columns (Varian, Lake Forrest, CA, USA). Eighty micrograms of peptides from each sample was labeled with TMT 6-plex reagents according to manufacturer instructions (Thermo Fisher Scientific). The samples were dried, resuspended in 0.1% trifluoroacetic acid, and desalted, and the peptide concentrations measured with the Pierce Quantitative Colorimetric Peptide Assay (Thermo Fisher Scientific). Small, equivalent volumes of samples were combined and a 500 ng aliquot was analyzed by mass spectrometry (below) to determine label incorporation percentage (>99%) and to estimate quantitative ratios between samples. The labeled samples were then mixed in equal amounts based on the quantitative ratios and separated by high pH reverse-phase HPLC through a Waters Xbridge 3.5 µm C18 column (4.6 × 15 cm) with a Dionyx UltiMate 3000 pump controlling a 38 min linear gradient from 4% to 28% acetonitrile and 0.5% triethylamine pH 10.7 (Wang et al., 2011). Seventy-five fractions were pooled by concatenation, dried, and resuspended in 5% acetonitrile and 0.1% formic acid, and the peptide concentrations were measured for 13 pools (Wang et al., 2011).

### Mass Spectrometry

Peptides (∼500 ng to 1 µg per pool) were separated on a 75 µm (inner diameter) fused silica capillary emitter packed with 22 cm of 2.5 μm Synergi Hydro-RP C18 (Phenomenex, Torrance, CA) coupled directly to a Dionex UltiMate 3000 RSLCnano System (Thermo Fisher Scientific) controlling a 180 min linear gradient from 3.2% to 40% acetonitrile and 0.1% formic acid at a flow rate of 300 nl per min. Peptides were electrosprayed at 2.4 kV into an Orbitrap Fusion Lumos Tribrid mass spectrometer (ThermoFisher) operating in data-dependent mode with positive polarity and using *m/z* 445.12003 as an internal mass calibrant. Quadrupole isolation was enabled and survey scans were recorded in the Orbitrap at 120,000 resolution over a mass range of 400–1,600 *m/z*. The instrument was operated in Top Speed mode using the multinotch MS^3^ method with a cycle time of 3 s (McAlister et al., 2014; Isasa et al., 2015). The automatic gain control (AGC) target was set to 200,000 and the maximum injection time was set to 50 ms. The most abundant precursor ions (intensity threshold 5,000) were fragmented by collision-induced dissociation (35% energy) and fragment ions were detected in the linear ion trap (AGC 10,000, 50 ms maximum injection). Analyzed precursors were dynamically excluded for 45 s. Multiple MS^2^ fragment ions were captured using isolation waveforms with multiple frequency notches and fragmented by high energy collision-induced dissociation (65% normalized collision energy). MS^3^ spectra were acquired in the Orbitrap (AGC 100,000; isolation window 2.0 *m/z*, maximum injection time 120 ms, 60,000 resolution scanning from 100–500 *m/z*).

### Peptide Matching and Statistics

Mass spectrometry data files were processed with Proteome Discoverer 2.1 (Thermo Fisher Scientific) which extracted MS^2^ spectra for peptide identification and MS^3^ spectra for peptide quantitation. MS^2^ spectra were searched with Mascot 2.5.1. (Perkins et al., 1999) against the *A. thaliana* protein database (TAIR10 with 35,386 records including splice variants) appended with a list of 172 sequences to detect common contaminants. Search parameters were for tryptic digests with two possible missed cleavages, fixed amino acid modification for chemically modified cysteine and labeled N-terminal and internal lysine (+57.021 Da, C; +229.163 Da, K), variable oxidized methionine (+15.995 Da, M), monoisotopic mass values, ± 10 ppm parent ion mass tolerance, and ±0.6 Da fragment ion mass tolerance. Peptide spectrum matches (PSM) were processed by Percolator (Kall et al., 2007) using delta Cn (0.05), strict false discovery rate (FDR) (0.01), relaxed FDR (0.05) and PEP (0.05) settings. Additional filters limited Mascot Ions scores (greater than or equal to 13) and PSM and peptide PEPs (strict 0.01; relaxed 0.05). Peptides were assigned to logical protein groups using parsimony. Proteins were quantified on summed signal-to-noise (S/N) ratios for each TMT channel for qualified PSMs for unique peptides (isolation interference < 25%, average reporter S/N > 8). The most confident centroid within 0.003 Da of the expected mass of the reporter ions was used. TMT signals were also corrected for isotope impurities (lot specific data provided by the manufacturer). Missing values were replaced with a minimum value. Matches to contaminants and decoys were removed from the dataset as were proteins with quantitative signal sums across all 6 channels <150 or proteins with less than 2 PSMs contributing to the qualified quantitative signal sum. Protein quantitative values for each channel were normalized and then scaled to 100 across the channels. Plotting the log2 fold changes after normalization (**Supplemental Figure S1A**) revealed a normal distribution. A t-test was used to measure significant differences and the Benjamini and Hochberg correction was applied to limit the FDR to 0.05. All proteins with an *FDR* < 0.01 and an abundance ratio (*sr45-1/*Col-0) > 1.20 or < 0.80 were defined as SR45-dependent differently accumulated proteins.

### RNA-Protein Expression Comparison

Comparisons were performed between the transcriptome data from our previous study (Zhang et al., 2017) and the proteome data from this study. All SR45-differentially regulated (SDR) RNAs identified by at least two independent pipelines (Tophat2, STAR and Lasergene v12) were pooled together to generate two RNA lists with either higher expression in Col-0 or with higher expression in *sr45-1* (**Supplemental Table S2**). Each of these two lists was compared with the respective protein list with either higher accumulation in Col-0 or with higher accumulation in *sr45-1*. Identities of SR45-ssociated RNAs (SARs), SR45-dependent alternative splicing (SAS) RNAs and SR45-dependent differentially accumulated (SDA) proteins found in inflorescences were also compared for overlap. All comparisons were performed using R.

### Functional Enrichment Analysis

PANTHER v14.0 (Mi et al., 2017) was used for GO term enrichment analysis. A list of proteins with greater accumulation in Col-0 and a list of proteins with greater accumulation in the *sr45-1* mutant were each submitted to STRING version 11.0 (Szklarczyk et al., 2015) for functional term enrichment analysis and visualization. All available evidence through STRING was used to define protein–protein associations. The evidence includes known interactions that were determined experimentally (from curated databases), predicted interactions (gene neighborhood, gene fusions, and gene co-occurrence), and other evidence (textmining, co-expression, and protein homology). A high confidence score of 0.700 was used as the minimum required score for filtering. Disconnected nodes in the network were not displayed due to the lack of evidence for their association with other proteins. Proteins belonging to enriched GO terms and/or KEGG Pathways that are highly relevant to RNA metabolism or known functions of SR45 were highlighted in different colors. All proteins with available KEGG IDs were mapped to their corresponding biological pathways using the KEGG mapping tool (www.genome.jp/kegg/).

### Predicted Protein Sequence and Structure Alignment

The amino acid sequences for SR45 (AtRNPS1, At1g16610), AtSAP18 (At2g45640) and AtACINUS (At4g39680) were used for sequence alignment with the sequences for animal ASAP complex protein model (PDB 4A8X) using ClustalW 2.1 (https://www.genome.jp/tools-bin/clustalw). The conserved sequences were then submitted to I-Tasser (Yang et al., 2015) for protein structure prediction. The predicted protein models were used to perform a structural alignment between *A. thaliana* and animal ASAP complex proteins using the PyMOL Molecular Graphics System, version 1.3 (Schrödinger, LLC).

### Total RNA Extraction and Real Time-qPCR

The RNeasy Plus Mini Kit (Qiagen) was used to extract RNA. About 5 μg of RNA from each sample was treated by DNase (ThermoFisher) followed by reverse transcription with Superscript IV (ThermoFisher). Real time-qPCR was performed using Power SYBR Master Mix (ThermoFisher) on a CFX96 machine (Bio-Rad). Expression levels were normalized to the expression of *GAPDH*. Primer sequences are listed in **Supplemental Table S1**.

### *SAP18* Cloning

The CDS of *SAP18* and the genomic *SAP18* (*gSAP*) sequences were amplified from either Col-0 inflorescence cDNA or genomic DNA using primers *SAP18ATGXhoI* and *SAP18nonstopKpnI*. The PCR products were inserted into *XhoI/KpnI* sites in the same GFP overexpression vector (pGlobug) as used before (Zhang and Mount, 2009) to create a *35S::SAP18CDS-GFP* or *35S::gSAP18-GFP* fusion, respectively. The overexpression cassettes *35S::SAP18CDS-GFP-NOS3*’ and *35S::gSAP18-GFP-NOS3*’ were isolated by *Not*I and cloned into a binary vector pMLBart, separately. All primers used in the cloning process are listed in **Supplemental Table S1**.

### Plant Transformation, Screening, and Verification of Transgenic Plants

DNA plasmids *pMLBart-35S::SAP18CDS-GFP-NOS3*’ and *pMLBart-35S::gSAP18-GFP-NOS3*’ were individually transformed into *Agrobacterium tumefaciens* GB3101 and used to transform *Col-0* and *sr45-1 *mutant plants by flower-dipping (Bent, 2000). All T1 plants were screened for *Basta *resistance by Finale (1:1,000 dilution) spray and examined for the GFP signal using a Nikon D-Eclipse C1 confocal microscope. At least 20 independent transgenic lines were selected for genotype confirmation using primers for the *SAP18-GFP *fusion (*SAP18ATGXhoI *and *GFPR *in **Supplemental Table S1**) and validated by confocal imaging using an Eclipse T*i* confocal microscope (Nikon).

### Quantification of GFP Signal Intensity in Transgenic Root Cells

Eight days old *35S::gSAP18-GFP* transgenic seedlings were used for quantifications of GFP signal intensity in root cells using an Eclipse T*i *confocal microscope (Nikon). For each root, a Z-stack was generated with 0.35 um per section for a total of 12 sections. The maximum signal was obtained by NIS Element (Nikon). Then the GFP signal intensity in nucleus and cytoplasm in each cell was quantified by ImageJ. A total of 15 cells per seedling were used for measurement in 3 seedlings. The average ratio of nucleus-to-cytoplasm GFP intensity per seedling was used for statistical analysis.

### Statistical Analysis

Normal distribution of samples was tested by Shapiro-Wilk normality test. For experiments that passed the normality test, one-way ANOVA followed by Tukey’s HSD test was performed when comparing more than two groups to each other. Unpaired Student t-test was used for two groups comparison. For experiments that did not pass the normality test, Kruskal-Wallis followed by post-hoc Dunn test was performed when comparing more than two groups to each other, Benjamini-Hochberg FDR method was used to calculate adjusted *p*-values.

## Results

### SR45 Modulates Proteins Functioning in RNA Metabolism

We previously compared RNA sequences from inflorescence from *A. thaliana* Col-0 and *sr45-1* mutants (Zhang et al., 2017). There were 358 differentially expressed RNAs (SDR). The SDR transcript gene ontologies (GO) did not explain flower development, but there was an elevated number of transcripts involved in immunity in the *sr45-1* mutants. The analysis also identified 542 SR45-dependent alternative splicing events (SAS) that, for the most part, did not overlap with gene expression changes. Again, GO analysis of these transcripts did not explain flower development, but rather revealed the breadth of transcripts that SR45 influences through alternative splicing. Immunoprecipitation, however, revealed a set of RNAs physically associated with SR45 (SAR) that included transcripts encoding nucleic acid binding proteins involved in the regulation of flowering, flower development, and embryo development in seeds. These results prompted us to investigate *sr45-1* mutant inflorescence at a proteomic level.

We extracted protein from the remaining bulked inflorescence tissue used for the RNA sequencing study. This included three biological replicates from Col-0 and the *sr45-1* null mutants. Tandem Mass Tag-based quantitative mass spectrometry was employed to generate quantifiable results. After searching the MS2 spectra against the *A. thaliana* protein database of 35,386 records in TAIR10, including splicing variants, a total of 58,962 peptides were determined from 101,605 peptide-spectrum matches (PSMs) (**Supplemental Figure S1B**). From these peptides, 10,120 *A. thaliana* proteins were identified (**Supplemental Figure S1B**). The quantification methods yielded 7,206 proteins with high-quality quantification information (**Supplemental Figure S1C**). On the basis of the quantified relative abundances of proteins, the three biological replicates of Col-0 closely clustered together and were distinct from the cluster of three biological replicates of the *sr45-1* mutant (**Supplemental Figure S1C**). This provides high confidence within the rigors of the experimental procedure and subsequent data analysis.

A total of 227 proteins exhibited statistically different accumulation with at least 20% fold change (**Supplemental Table S3**). Among these proteins was SR45 which had a 93% decrease in accumulation in *sr45-1*. It has been confirmed that *sr45-1* is a T-DNA knock-out, and it does not produce a full-length transcript. However it does produce a truncated transcript at 8% the rate of the full-length transcript in wild type (Ali et al., 2007). It would be unlikely for this truncated transcript to produce a viable peptide because it lacks a stop codon and 3’ UTR for polyadenylation. The reason why SR45 did not appear to be absent in the mutant is because TMT signal reporting is relative, not absolute, and because signal values, even small ones, are normalized and scaled for analysis (Rauniyar and Yates, 2014). Nonetheless, SR45 exhibited the greatest decrease of all proteins measured, which is consistent with our expectation.

**Figure 1:**
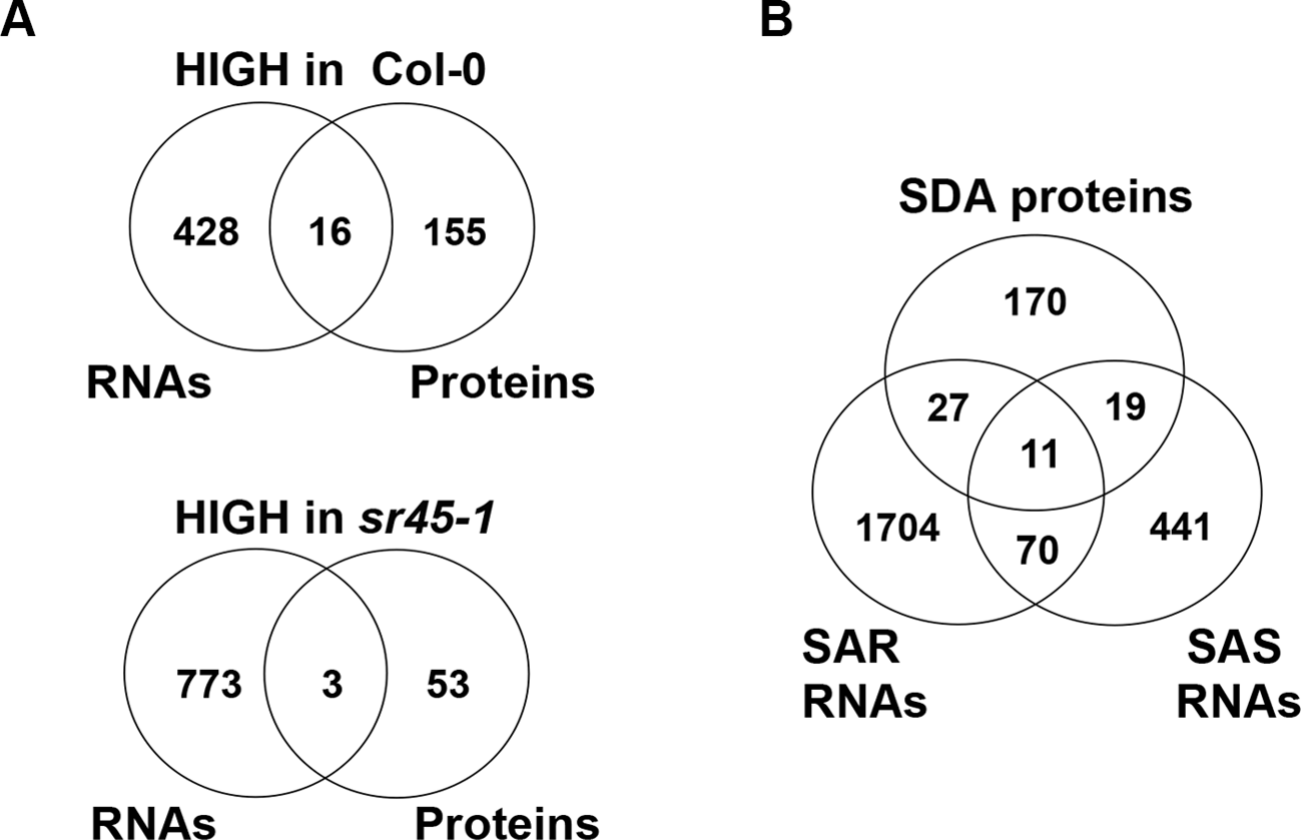
An RNA-protein expression association study. **(A)** A comparison between SR45-differentially regulated RNAs and SR45-dependent differentially accumulated (SDA) proteins; **(B)** a comparison between SR45-dependent differentially accumulated (SDA) proteins, SR45-dependent alternatively spliced (SAS) RNAs and SR45-associated RNAs (SARs), published previously (Zhang et al., BMC Genomics, 2017).

The other 226 proteins, defined as SR45-dependent differentially accumulated (SDA) proteins, were mostly distinct from the SDR RNAs identified from the same tissue (Zhang et al., 2017). In order to increase the coverage of SDR RNAs, we combined all SDR RNAs identified by any two of the three independent pipelines (Tophat2, STAR and Lasergene v12) to create larger datasets of 444 SR45-upreguated RNAs and 776 SR45-downregulated RNAs (**Supplemental Table S2**), whereas in our previous methods, SDR RNAs were determined only when all three pipelines agreed (Zhang et al., 2017). Even with the larger SDR RNA datasets, only 19 (8% of 227) of SDA proteins overlapped (**Figure 1A**, **Table 1**). However, 57 (25% of 227) of SDA proteins were found to be SAR products, SAS products, or both (**Figure 1B**), although their RNAs were not differentially expressed. In the *sr45-1* mutant, the splicing pattern of 11 SARs was altered and there was a lower steady state level of their corresponding protein products (**Figure 1B**, **Table 2**). It is quite possible that after SR45 binding to RNA targets, the splicing pattern of these RNAs was influenced, which provided the template for the formation of protein products. Some of the RNAs for the SDA proteins mentioned above have been confirmed for differential expression and/or alternative splicing changes (Zhang et al., 2017). Most of these 11 genes code for enzymes, one of which is a jmjC domain-containing lysine-specific histone demethylase (IBM1, **Table 2**). IBM1 reduces histone H3K9 methylation and prevents CHG hypermethylation in active genes in *A. thaliana* and *Populus* (Miura et al., 2009; Fan et al., 2018). Here, IBM1 protein had reduced accumulation (72.5% of Col-0) in the *sr45-1* mutant, which suggests that at least some of its target genes could be hypermethylated and therefore expressed at a lower level in the *sr45-1* mutant. Indeed, genes encoding 57 proteins, one-third of 171 proteins that have decreased abundance in the *sr45-1* mutant, were found to be hypermethylated in the *ibm-1* loss-of-function mutant (**Supplemental Table S4**) (Miura et al., 2009). Five of them are SARs, SAS products and code for SDA proteins (**Table 2**). These hypermethylated regions were mostly found in the gene body, not necessarily in the promoter regions (Miura et al., 2009). Nevertheless, the comparison between RNA and protein from the same tissues suggests that the majority of the SDA protein accumulation changes did not arise from transcriptional changes.

**Table 1:** A summary of genes that are differentially expressed in an SR45-dependent manner at both RNA and protein levels. Their identity as either SAS or SAR genes is also listed below.

AGI	Gene names	RNA FC	Protein FC	SAS/SAR
		(*sr45-1/Col-0*)	(*sr45-1/Col-0*)	
AT1G11930	At1g11930/F12F1_20	0.476	0.348	SAS
AT1G16610	SR45	0.156	0.072	SAS/SAR
AT1G20120	GDSL esterase/lipase	0.411	0.276	SAS
AT1G21650	Protein translocase subunit SECA2, chloroplastic	0.385	0.587	
AT2G21385	ATCGLD11, BFA3, BIOGENESIS FACTORS REQUIRED FOR ATP SYNTHASE 3, CGLD11	0.436	0.268	
AT2G21590	APL4	0.459	0.543	
AT2G22400	ATTRM4B, TRM4B	0.352	0.498	
AT2G27880	AGO5, ARGONAUTE 5, ATAGO5	0.344	0.437	SAR
AT3G14150	Peroxisomal (S)-2-hydroxy-acid oxidase GLO3	0.416	0.591	SAS
AT3G42850	ARA2, ARABINOKINASE 2	0.456	0.611	SAR
AT4G38350	Patched family protein	0.468	0.332	SAR
AT4G39460	S-adenosylmethionine carrier 1, chloroplastic/mitochondrial;SAMC1	0.470	0.461	
AT5G13380	Auxin-responsive GH3 family protein	0.464	0.782	
AT5G23960	Alpha-humulene/(-)-(E)-beta-caryophyllene synthase;TPS21	0.351	0.629	
AT5G45140	NRPC2, NUCLEAR RNA POLYMERASE C2	0.496	0.569	SAR
AT5G63810	ATBGAL10, BETA-GALACTOSIDASE 10, BGAL10	0.456	0.442	
AT2G43620	Chitinase family protein	3.358	3.472	
AT4G25000	ALPHA-AMYLASE-LIKE, AMY1, ATAMY1	2.353	1.852	
AT5G25450	Cytochrome bd ubiquinol oxidase, 14kDa subunit	3.581	1.385	

**Table 2:** A summary of genes of which their RNAs are identified as SR45-associated (SAR) and SR45-dependent alternatively spliced (SAS), and of which their proteins are differentially accumulated in an SR45-dependent manner. The RNA for these proteins is not differentially expressed in the *sr45-1* mutant (Zhang et al., 2017).

AGI	Gene names	Protein FC	Hypermethylated gene in *ibm1 *mutant
		(*sr45-1/*Col-0)	
AT1G16610	Serine/arginine-rich splicing factor SR45;SR45;ortholog	0.072	
AT1G65540	LETM1-like protein;At1g65540;ortholog	0.244	Yes
AT5G13980	Alpha-mannosidase; At5g13980;ortholog	0.350	Yes
AT3G10160	Folylpolyglutamate synthase;FPGS2;ortholog	0.448	Yes
AT2G30170	Probable protein phosphatase 2C 26;At2g30170;ortholog	0.457	
AT4G30310	FGGY family of carbohydrate kinase;At4g30310;ortholog	0.505	
AT5G13690	Alpha-N-acetylglucosamini dase;CYL1;ortholog	0.515	Yes
AT4G10060	Non-lysosomal glucosylcerami dase; At4g10060;ortholog	0.535	
AT4G10030	Hydrolase, alpha/beta fold family protein; T5L19.160;ortholog	0.633	
AT3G07610	Lysine-specific demethylase JMJ25;JMJ25;ortholog	0.725	Yes
AT1G01710	Acyl-CoA thioesterase family protein;At1g01710;ortholog	0.741	

**Figure 2:**
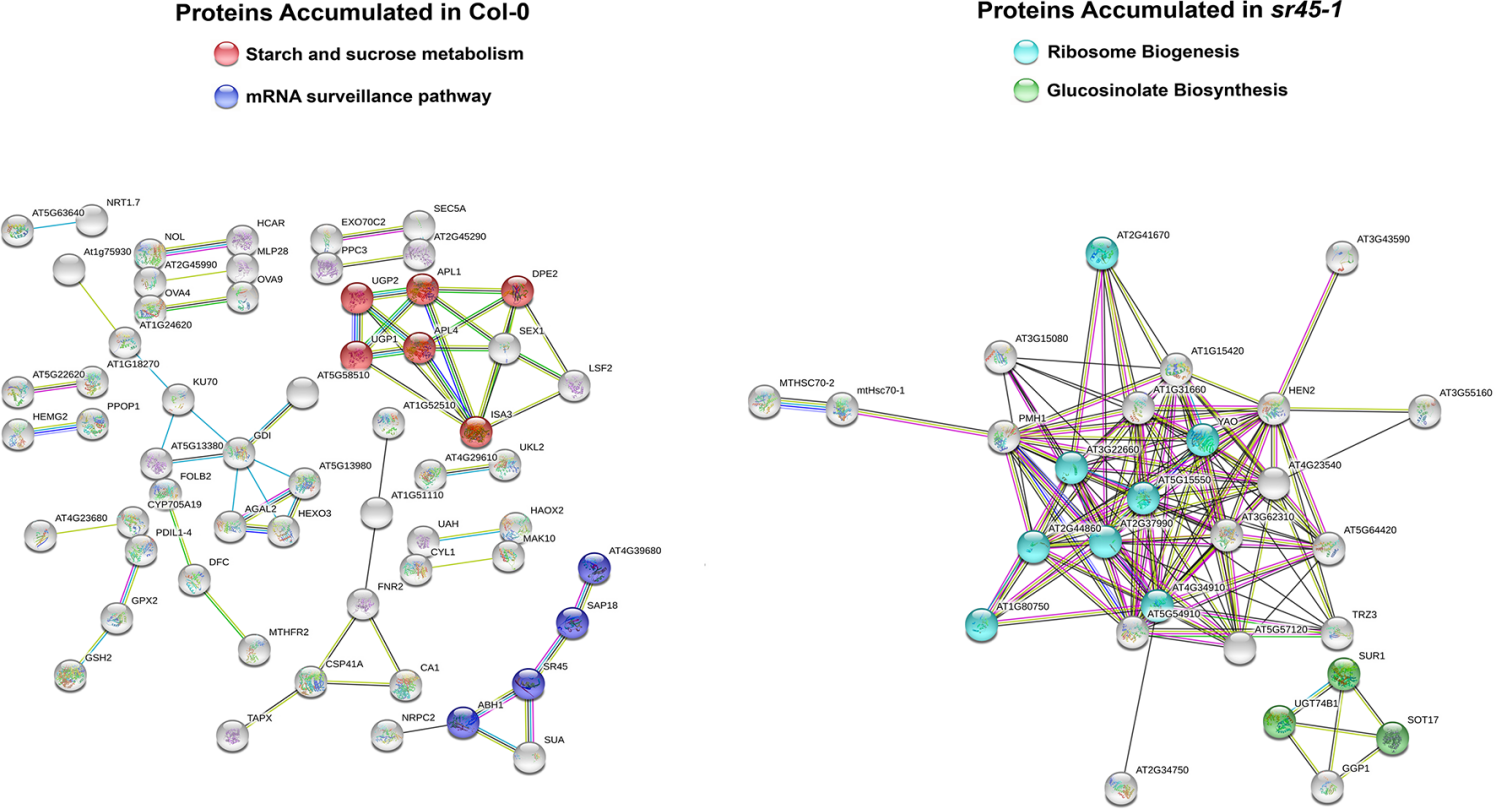
Functional enrichment analyses using STRING version 11.0. Left: Proteins with higher accumulation in Col-0 enriched in two sample pathways as highlighted with two different colors. Right: Proteins with higher accumulation in *sr45-1* enriched in two sample pathways as highlighted with two different colors. Each sphere represents a protein node in the network. Each edge presents an existing piece of support evidence collected by STRING. All evidence used to build the protein network was filtered with a high confidence of 0.7000.

Bioinformatics programs PANTHER (Mi et al., 2017), KEGG (www.genome.jp/kegg/) and STRING (Szklarczyk et al., 2015) were used to gain insights to the SDA protein functions. The 171 proteins with decreased accumulation in the sr45-1 mutant were overrepresented in starch and sucrose metabolism and mRNA surveillance pathways, whereas the 56 proteins with increased accumulation appeared to be overrepresented in ribosome biogenesis and glucosinolate biosynthesis pathways (**Figure 2**, **Supplemental Table S5**).

### mRNA Surveillance

The mRNA surveillance pathway includes RNA quality control through the EJC and the degradation of aberrant RNAs. It identifies PTC-containing mRNAs and prevents them from being used for translation (Popp and Maquat, 2013). Four proteins in this pathway had significantly less accumulation in the *sr45-1* mutant compared to Col-0 (**Supplemental Table S3**). One was SR45, as expected. The next was AtSAP18, exhibiting the next greatest fold decrease of 0.128 (an 87% reduction, see **Supplemental Figure S2A**). This suggests that the mutational absence of SR45 led to a near absence of AtSAP18. The other two proteins, AtACINUS and ABA HYPERSENSITIVE 1 (ABH1), had fold reductions of 0.664 and 0.796 respectively (**Supplemental Figure S2A** and **Supplemental Table S3**). SR45, AtSAP18, and AtACINUS appear to be the counterparts of the ASAP core proteins in animals. Meanwhile, ABH1 functions as a cap-binding protein [cap-binding protein 80 (CBP80)] and stabilizes CBP20 in the nucleus when binding to the 7-methylguanosine cap at the 5’ end of mature mRNAs (Kierzkowski et al., 2009). When the CBP80/20 complex binds to the 5’-end of capped target transcripts, it plays dual roles by directly influencing alternative splicing, mostly at the 5’ splice site of the first intron, and pri-miRNA processing (Laubinger et al., 2008; Raczynska et al., 2010). Hence, the results suggest that the absence of SR45 led to the substantial reduction of other ASAP core proteins, likely through complex instability. This might have a substantial effect on associated processes covering different steps of RNA metabolism from transcription to alternative splicing. It is possible that these proteomic differences contributed to the transcriptome level differences between Col-0 and the *sr45-1* mutant reported previously (Zhang et al., 2017) and to the other protein differences that follow.

### Ribosome Biogenesis

The biogenesis of ribosomes produces the machinery for translation. During ribosome biogenesis, both ribosome RNAs (rRNAs) and ribosome proteins need to be mature and assembled. This is an energy-consuming process and requires the coordination and regulation control among RNA polymerases I, II, III, and the splicing of introns (Nerurkar et al., 2015). A total of 8 proteins in the ribosome biogenesis pathway exhibited moderately elevated fold increase (1.214–1.450) in the *sr45-1* mutant. They are involved in rRNA processing, maturation and ribosome assembly (**Supplemental Table S5**). Although the change in each of them was relatively mild, the aggregated catalytic outcome in ribosome biogenesis could be more notable than each individual increase represents. Taken with RNA surveillance, SR45 seems to have a preference in modulating the abundance of protein factors functioning in more than one aspect of RNA metabolism. As of now, however, there are no clear explanations for how the loss of SR45 could cause the increase in ribosome biogenesis proteins in inflorescence.

### Plant Defense

Proteins with greater accumulation in the *sr45-1* mutant included several that may be related to immunity to disease. Three proteins, UGT74B1 (At1g24100), SUR1 (At2g20610) and SOT17 (At1g18590), had fold increases ranging from 1.272 to 1.841 in the *sr45-1* mutant (**Supplemental Table S5**). These three proteins catalyze the last three consecutive steps of the glucosinolate biosynthetic pathway (Grubb and Abel, 2006). Glucosinolates are used in plant defense (Grubb and Abel, 2006; Bednarek et al., 2009). An aggregated increase of all three of the enzymes could result in more glucosinolate production in the *sr45-1* plants. Meanwhile, the protein with the greatest increase (a fold increase of 3.472) in the mutant was an endochitinase, while two others that also significantly increased in the mutant were catalase (increased to 1.760) and peroxidase (increased to 1.750) (**Supplemental Table S3**). These three enzymes often increase in accumulation during pathogen attack or during cell wall metabolism. These results agree with increased pathogen resistance, increased reactive oxygen species, and increased cell wall callose deposition in *sr45-1* mutants (Zhang et al., 2017).

### The Structure of the *Arabidopsis* ASAP Complex Closely Resembles the Animal Core ASAP Complex

In animal models, the ASAP complex has three core components, RNPS1, SAP18, and ACINUS. A crystal structure of the core ASAP complex (4A8X) is available in the Protein Data Bank. It comprises the conserved RNA Recognition Motif (RRM) in human RNPS1 (HsRNPS1), a ubiquitin-like (UBL) domain in mouse SAP18 (MmSAP18), and a RNPS1-SAP18-binding (RSB) motif in *Drosophila* ACINUS (DmACIN) (Murachelli et al., 2012). To examine how closely these conserved domains in the three *A. thaliana* proteins resemble those in their animal counterparts, the corresponding amino acid segments were used for pair-wise alignment (**Supplemental Figure S3**). SR45 protein has two RS domains flanking an RRM, which is distinct from all other *A. thaliana* SR proteins (Barta et al., 2010); rather, it resembles the domain structure of RNPS1 (Zhang and Mount, 2009). The SR45 RRM sequence had 36.4% amino acid identity and 73.9% similarity to the HsRNPS1 RRM (**Supplemental Figure S3A**). The AtSAP18 fragment sequence for the UBL domain had 53.6% amino acid identity and 80.0% of similarity to the MmSAP18 UBL domain (**Supplemental Figure S3B**). The AtACINUS sequence RSB motif had 64.0% amino acid identity and 84.0% similarity to the DmACINUS RSB sequence (**Supplemental Figure S3C**). The predicted domain structure for the *A. thaliana* ASAP complex core proteins aligned with their animal counterparts in 4A8X. Specifically, SR45 and AtSAP18 aligned closely with HsRNPS1 and MmSAP18, respectively (**Supplemental Figure S2B**). The alignment of the RNPS1 RRM structure yielded an all-atom root-mean-square deviation of atomic positions (RMSD) of 2.350 Å (**Supplemental Figure S2C**). The predicted SR45 RRM protein model lacked 4 small beta sheets compared to HsRNPS1 RRM, which seemed to have little effect on the overall structure of the RRM itself. The alignment of the SAP18 UBL domain structure yielded an all-atom RMSD of 4.300 Å (**Supplemental Figure S2C**). In comparison to MmSAP18, AtSAP18 had a different small alpha helix. This caused minimal disturbance in the overall UBL domain structure. The alignment of ACINUS yielded an all-atom RMSD of 6.888 Å (**Supplemental Figure S2C**). However, the predicted AtACINUS RSB structure did not align as well with DmACINUS RSB. In this small region, AtACINUS was missing 2 small beta sheets compared to DmACINUS, which seemed to influence the orientation of the overall RSB structure. Nevertheless, both sequence and structure alignments supported the hypothesis that the *A. thaliana* ASAP complex is orthologous to the ASAP complex found in animals. In the rest of the text, we will refer to AtSAP18 and AtACINUS as SAP18 and ACINUS.

### SR45 Maintains the Wild-Type Level of the ASAP Complex Core Component Proteins

**Figure 3:**
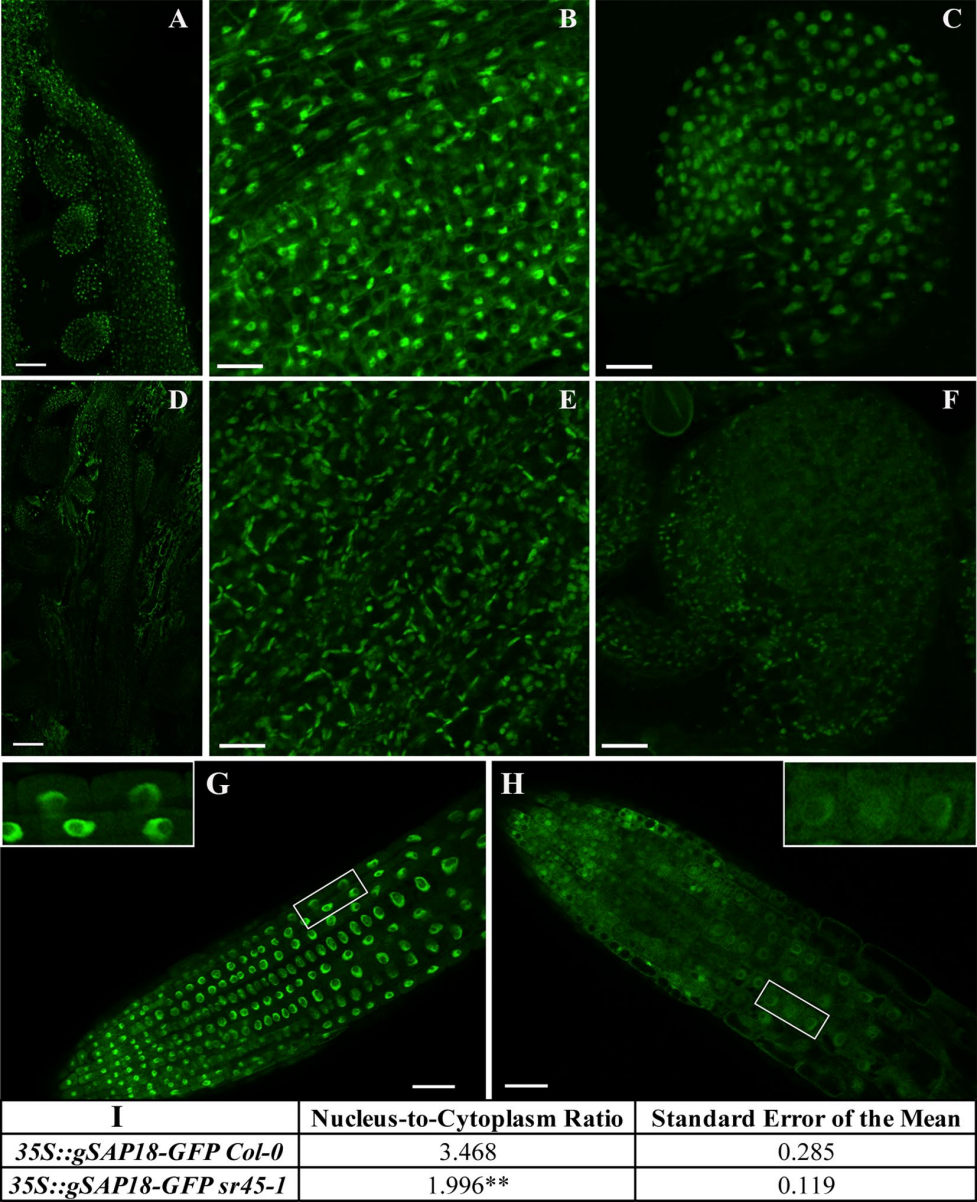
A comparison of SAP18-GFP expression between Col-0 and *sr45-1*. **A-C** and **G** represent Col-0 transgenic; **D-F** and **H** represent *sr45-1* transgenic. **(A)** and **(D)**: carpel containing ovule inside with scale bars = 50 µm. **(B)** and **(E)**: a close-up view of carpel cells with scale bars = 25 µm. **(C)** and **(F)**: a close-up view of ovules with scale bars = 25 µm. **(G)** and **(H)**: root tip with scale bars = 50 µm. The inserts showed a close-up view of root cells in the boxed area. **(I)**: qualifications of root GFP signal intensity in nucleus vs. cytoplasm. A total of 15 cells were measured per seedling. Three seedling were used for student t-test **: p < 0.01.

**Figure 4:**
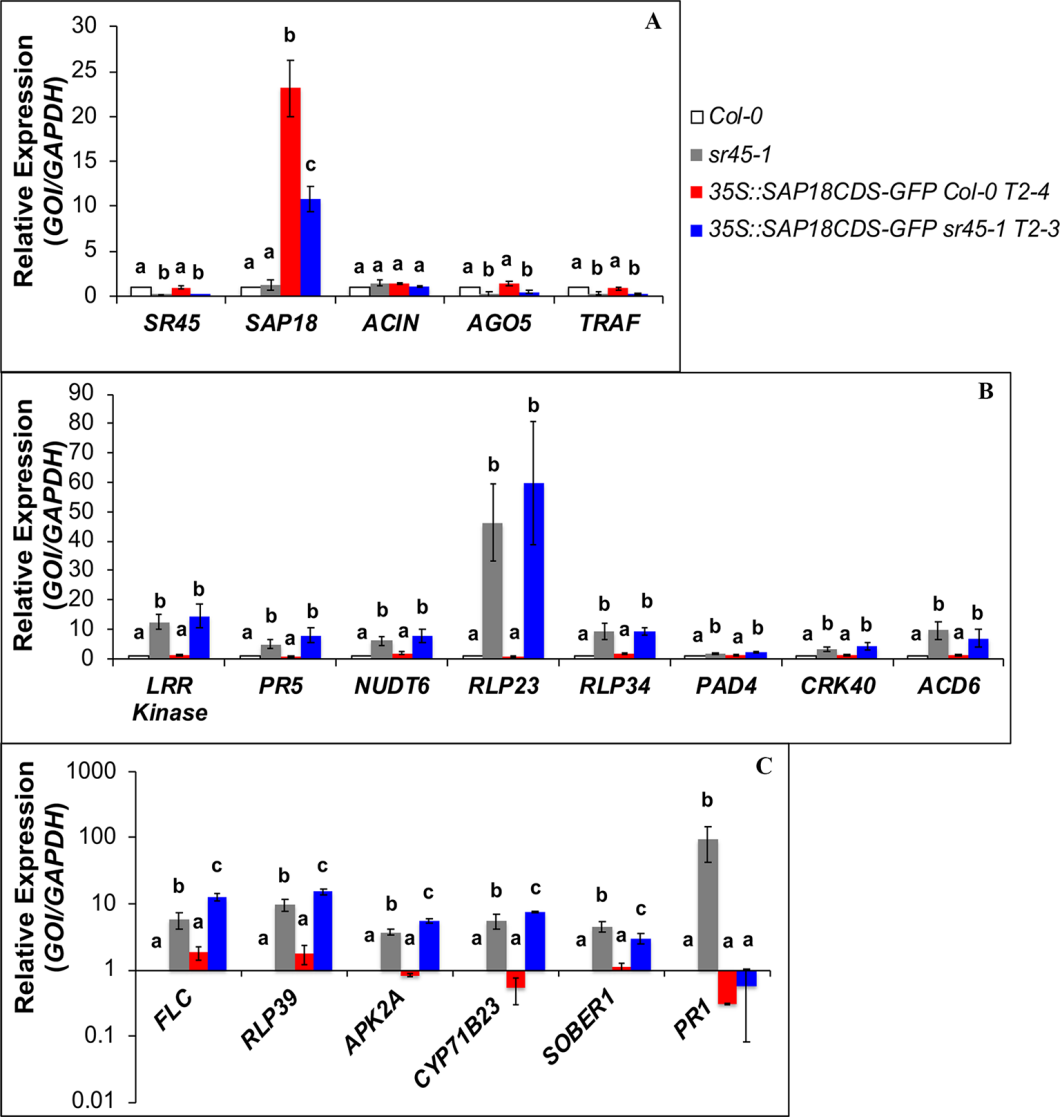
RT-qPCR on SDR genes in Col-0, *sr45-1, 35S::SAP18CDS-GFP* Col-0 and *35S::SAP18CDS-GFP sr45-1*. **(A)** SR45-upregulated genes; **(B)** SR45-downregulated genes that are not affected by SAP18-GFP overexpression; **(C)** SR45-downregulated genes that are affected by SAP18-GFP overexpression. One-way ANOVA followed by Tukey HSD was used for statistical analysis. Letters a–c represent different levels of statistical significance with *p* < 0.05. *n* = 3. Error bars represent standard deviation.

To evaluate the function of SAP18 with respect to SR45, we produced transgenic plants overexpressing an in-frame fusion of g*SAP18-GFP* in Col-0 and in the *sr45-1* mutant (**Figure 3**). In the inflorescence tissue, bright and distinct nucleoplasmic GFP signal was detected in carpels and ovules in the Col-0, while only very dim nucleoplasmic GFP signal was detected in *sr45-1* in a few carpel cells overshadowed by chloroplasts. If it was not for the chloroplasts, the *sr45-1* ovule would have been barely noticeable (**Figures 3A–F**). To visualize the subcellular distribution of SAP18-GFP more clearly without the strong background from chloroplasts, root tips of seedlings were compared between the transgenic Col-0 and *sr45-1* lines. In the transgenic Col-0 root tip, the SAP18-GFP signal was prominent and strong in the nucleoplasm, and it was much lower and diffused in the cytoplasm of every cell in the meristematic zone. In the transgenic *sr45-1* mutant, however, the overall SAP18-GFP signal was much weaker and more diffused. More specifically, there was much less nuclear SAP18-GFP in the transgenic *sr45-1* mutant root compared to the transgenic Col-0, even though the intensities of their cytosolic SAP18-GFP signals were similar (**Figures 3G, H**). A statistically significant 1.7-fold reduction in nucleus-to-cytoplasm ratios of GFP signal intensity (3.468 in Col-0 background vs. 1.996 in *sr45-1* background) was observed when comparing *35S::gSAP18-GFP* transgenic seedlings in the Col-0 background to transgenic seedlings in the *sr45-1* background (**Figure 3I**). RT-qPCR results revealed that there was no significant difference in the transcript for both the *SAP18-GFP* transgene and the endogenous *SAP18* between the transgenic *sr45-1* and transgenic Col-0. (**Supplemental Figure S4**). Thus, these data support the notion that SR45 protein is required to maintain nuclear SAP18 protein at the wild-type level, a hypothesis corroborated by the proteomics data. RT-qPCR also revealed that the expression of *ACINUS* was unchanged in the *sr45-1* mutant (**Figure 4A**), indicating SR45’s possible role in a protein control for ACINUS as well.

### SAP18 Participates in the Suppression of a Subset of SR45 Differentially Regulated RNAs

In our prior study, we identified 358 SDR RNAs differentially expressed in *sr45-1* inflorescence (Zhang et al., 2017). To understand whether SAP18 is involved in the regulation of SDR RNAs and RNAs for ASAP core components, RT-qPCR was performed in Col-0, *sr45-1*, and the *SAP18-GFP* overexpression lines. Although there was no statistically significant difference in the RNA level of *SAP18* between Col-0 and the *sr45-1* mutant, there was a substantial 10–22 fold increase in the overall *SAP18* RNA, including the *SAP18-GFP* transgene and endogenous *SAP18*, due to *SAP18-GFP* transgenic overexpression (**Figure 4A**). This dramatic change, however, did not seem to affect the expression of *SR45* and *ACINUS* (**Figure 4A**). Neither did it change the expression pattern for two previously confirmed SR45-upregulated SDR RNAs, *AGO5*, and *TRAF* (**Figure 4A**), nor eight SR45-downregulated SDR genes known to play roles in plant immunity (**Figure 4B**). However, the expressions of defense genes *SOBER1* and *PR1* decreased or returned to a level observed in non-transgenic Col-0 (**Figure 4C**), while the expressions for three other defense–response genes, *RLP39, APK2A*, and *CYP71B23*, increased (**Figure 4C**). These findings suggest that SAP18 is involved in the control of the expression of a subset of SDR defense genes.

### Overexpression of SAP18 Further Delays Flowering in the *sr45-1* Mutant

**Figure 5:**
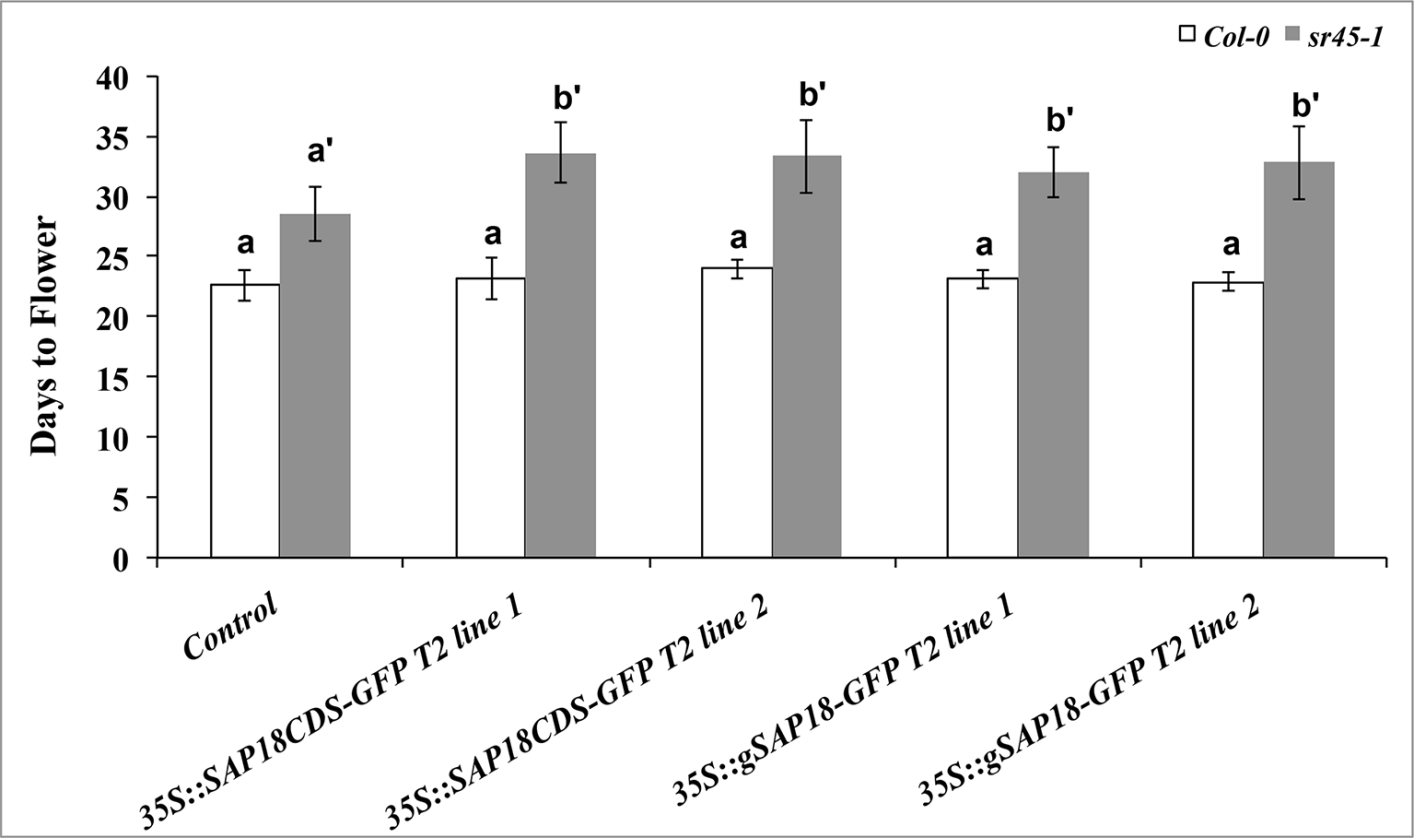
A comparison of flowering time among different genotypes grown under long-day condition (L:D = 16:8). The number of days before bolting is used to indicate days to flower. Transgenic lines were compared with their corresponding controls (Col-0 or *sr45-1*), respectively. Kruskal-Wallis test followed by post-hoc Dunn test was used for statistical analysis. Benjamini-Hochberg FDR method was used to calculate adjusted *p*-values. Letter a represents statistical level of Col-0 and its transgenic lines; letters a’ and b’ represent different levels of statistical significance for *sr45-1* and its transgenic lines. *n* = 10. *FDR* < 0.05. Error bars represent standard deviation.

The *sr45-1* mutant is delayed in flowering, and it has increased expression of *FLC*, a flowering suppressor (Ali et al., 2007). Interestingly, overexpression of *SAP18-GFP* in the *sr45-1* mutant resulted in a modest but statistically significant increase in the RNA levels of *FLC* compared to the non-transgenic *sr45-1* mutant (**Figure 4C**). To determine the physiological effect of this, we evaluated the flowering time of transgenic plants with the *SAP18CDS-GFP* or the *gSAP18-GFP* gene fusion versions. Transgenic Col-0 plants overexpressing either *SAP18-GFP* transgene versions had no detectable difference in flowering time compared to non-transgenic Col-0. Surprisingly, transgenic *sr45-1* plants overexpressing either of the two *SAP18-GFP* transgenes were further delayed in flowering by 4 days compared to the non-transgenic *sr45-1* parent which already has a late flowering phenotype (**Figure 5**). This phenotype was consistent in independent transgenic lines. These observations suggest that SR45 may be required to control SAP18 in flowering time suppression. A recent study has shown that all ASAP core complex proteins were associated with the vernalization complex *via* VAL1 to induce transcriptional silencing at the *FLC* locus (Questa et al., 2016). Therefore, it is possible that SR45 and SAP18 work together to achieve an optimal suppression of *FLC* and the initiation of flowering.

## Discussion

RNA metabolism is a complex and concerted process that requires both protein factors and RNA substrates. Our previous study described SR45-dependent RNA level changes in the inflorescence tissue (Zhang et al., 2017). In this study, using the same batch of inflorescence tissue as used in the transcriptome study, we found that proteins differentially accumulated in the *sr45-1* mutant likely participate in different steps of RNA metabolism, especially mRNA surveillance (**Figure 2** and **Supplemental Table S3**). This provides a protein context in RNA metabolism for the transcriptome difference we described before (Zhang et al., 2017). The fact that there is little overlap between the differentially expressed RNAs and differentially accumulated proteins strongly suggests that most SDA proteins are not direct products of the differentially expressed RNAs. Alternative splicing does not sufficiently explain this discordance either. Rather, the proteins coexisted with the differentially expressed RNAs in the same inflorescence tissue and are likely a product of transcriptional and post-translational regulation that arises from the loss of the SR45 protein.

Despite the evidence that SR45 is a splicing regulator, there is also evidence that SR45, but not other splicing factors, may regulate RdDM-mediated DNA methylation *via* unknown mechanisms (Ausin et al., 2012). Our data indicates that hypermethylation in gene body of IBM targets may be one such mechanism (**Supplemental Table S4**). In our study, all core proteins of a conserved ASAP complex, SR45, SAP18, and ACINUS, exhibited a significantly lower level of accumulation in the *sr45-1* mutant. Their orthology to the ASAP complex proteins in animal models, shown here through amino acid sequence homology and predicted protein domain structures (**Supplemental Figures S2** and **S3**), supports the hypothesis that SR45 not only functions as a splicing regulator to affect pre-mRNA splicing, but can influence transcriptional control *via* associations with other proteins that are not splicing factors (Song and Galbraith, 2006; Hill et al., 2008; Zhang and Mount, 2009; Day et al., 2012; Xing et al., 2015; Questa et al., 2016; Deka and Singh, 2017; Zhang et al., 2017). Such an example is its interaction with SAP18 in the ASAP complex.

Understanding the effect of SR45 on SAP18 is key to revealing the function of the ASAP complex on affected processes. Although there is limited knowledge on the role of SR45 in the *A. thaliana* ASAP complex, SR45 seems to maintain the wild-type level of SAP18 protein in the nucleus. Without SR45, the level of nuclear SAP18 protein is drastically decreased (**Figure 3**). This could have a broad impact on processes not directly related to splicing. Since SAP18 recruits HDACs to chromatin for gene silencing, less nuclear SAP18 protein could reduce the abundance of HDACs on the affected loci leading to a leaky gene expression. Specifically, a transcriptional repressor VAL1 can interact with the ASAP complex and promote HDA19 docking at the *FLC* locus to silence *FLC* (Questa et al., 2016). The RNA level of *FLC* was slightly higher in an HDAC mutant *hda19-1*, an *sr45* T-DNA insertion mutant and a *sap18* T-DNA insertion mutant. We overexpressed *SAP18-GFP* in the *sr45-1* mutant and observed a correlation between the increased expression level of *FLC* and increased time to flower compared to the non-transgenic *sr45-1* mutant (**Figures 4C** and **5**). It is unexpected to see that the overexpression of SAP18-GFP protein did not lead to a stronger suppression of *FLC* expression. On the contrary, it exacerbated the effect of the *sr45-1* mutation on *FLC* and caused a further delay in flowering. Together, these results imply that SR45 is required for a wild-type level of SAP18 accumulation in the nucleus and that FLC-regulated flowering time is controlled by the nuclear presence of two components of the ASAP complex.

The requirement of SR45 for proper SAP18 functions can also be seen in the expression of a subset of RNAs involved in defense that were previously found to have increased expression in *sr45-1* mutants (**Figure 4C**). This subset consists of five plant defense genes, among which is *PR1*, a marker for activation of the salicylic acid signaling pathway (Gaffney et al., 1993; van Loon et al., 2006). The expression of *PR1* RNA is returned to even lower level than in Col-0 by overexpressing *SAP18-GFP* in the *sr45-1* mutant, which indicates a possible reversal of the elevated SA pathway. Although enzymes for glucosinolate biosynthesis had higher accumulation in the *sr45-1* mutant to support the notion of a stronger immunity in the *sr45-1* mutant, it is unclear whether there is an actual change in glucosinolate production in the *sr45-1* mutant, and whether overexpressing *SAP18-GFP* would have any impact on the level of these enzymes and defense response. Future experiments in these areas will be necessary to answer the questions mentioned above.

In conclusion, this study provides evidence for the pleiotropic effects of SR45 on protein expression in *A. thaliana* inflorescence with an emphasis on different aspects of RNA metabolism. In addition to being a main component of the ASAP complex, SR45 also regulates the other two ASAP component proteins to achieve optimal transcriptional suppression of substrate target genes. Our findings also provided evidence for a new explanation beyond alternative splicing for the regulation of SR45-suppressed RNAs. Further studies on histone modification at SAP18-targeted loci will help elucidate whether and how a conserved ASAP complex functions to regulate these genes and proteins during flowering.

## Data Availability

The RNA-seq datasets used for RNA-protein comparison for this study can be found as a BioProject (PRJNA382852) in the NCBI Sequence Read Archive (SRA) [https://www.ncbi.nlm.nih.gov/bioproject/PRJNA382852/]. The mass spectrometry data files can be retrieved from massive.ucsd.edu (MSV000083728). All the peptide-spectrum match for the mass spectrometry data is available in **Supplemental Table S6**.

## Author Contributions

X-NZ conceived, designed, conducted, and supervised the study. HB, WG, and BC performed the quantitative proteomics experiment and subsequent statistical analyses. SC, TR, and X-NZ performed bioinformatics analysis and functional enrichment analysis. SC, TR, AH, LM, and JP conducted the study. X-NZ, SC, TR, AH, and BC wrote the paper.

## Funding

This work was supported by National Science Foundation (DBI-1146300 to XZ), USDA-ARS to BC, and research funds in Department of Biology at St. Bonaventure University.

## Conflict of Interest Statement

The authors declare that the research was conducted in the absence of any commercial or financial relationships that could be construed as a potential conflict of interest.
