# Supplementary figures and images for: Quantitative Proteomics Reveals a Role for SERINE/ARGININE-Rich 45 in Regulating RNA Metabolism and Modulating Transcriptional Suppression via the ASAP Complex in Arabidopsis thaliana
Source: Front Plant Sci. 2019 Sep 19;10:1116. doi: 10.3389/fpls.2019.01116 (PMC6761909; doi:10.3389/fpls.2019.01116)

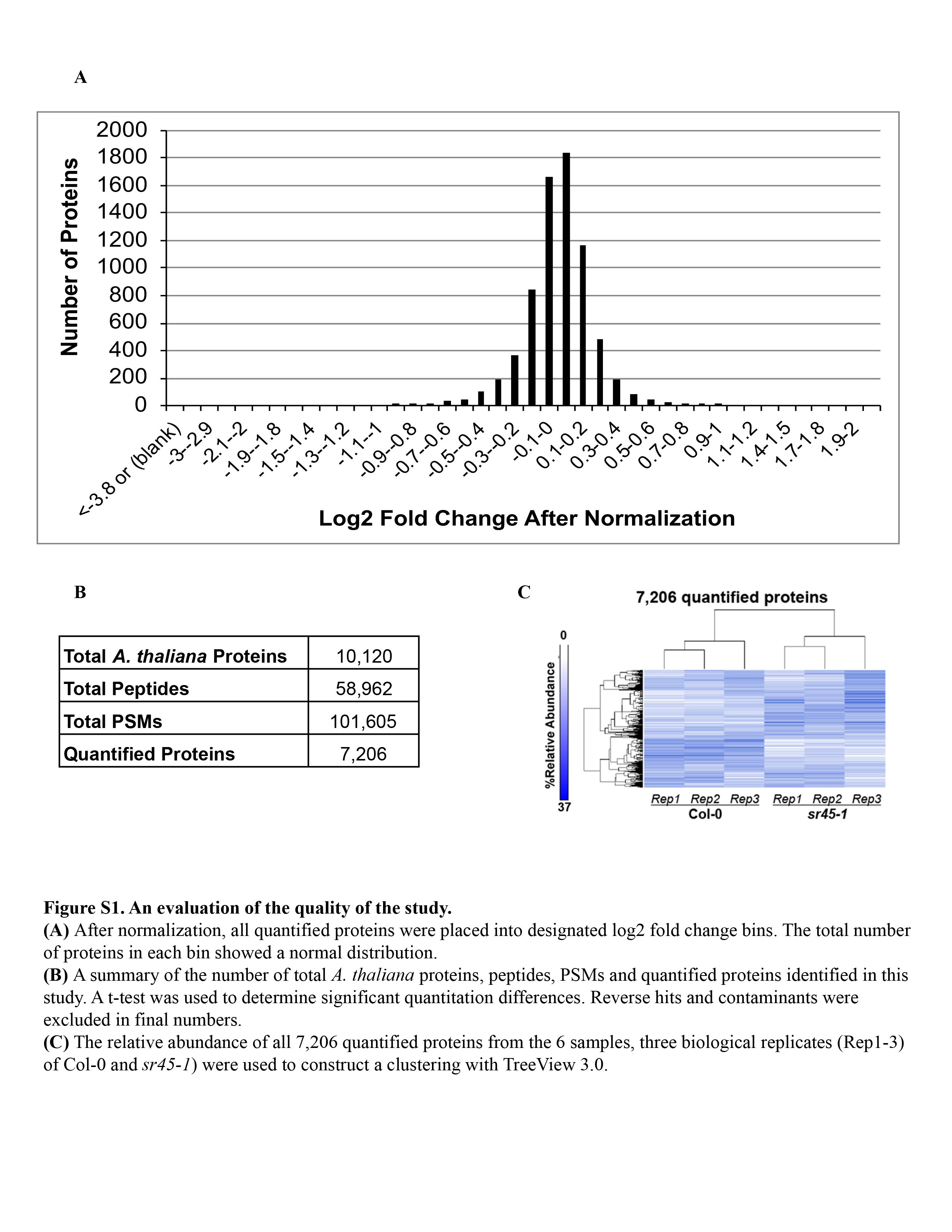

Supplement: Supplementary file 1 [file Image_1.tif]

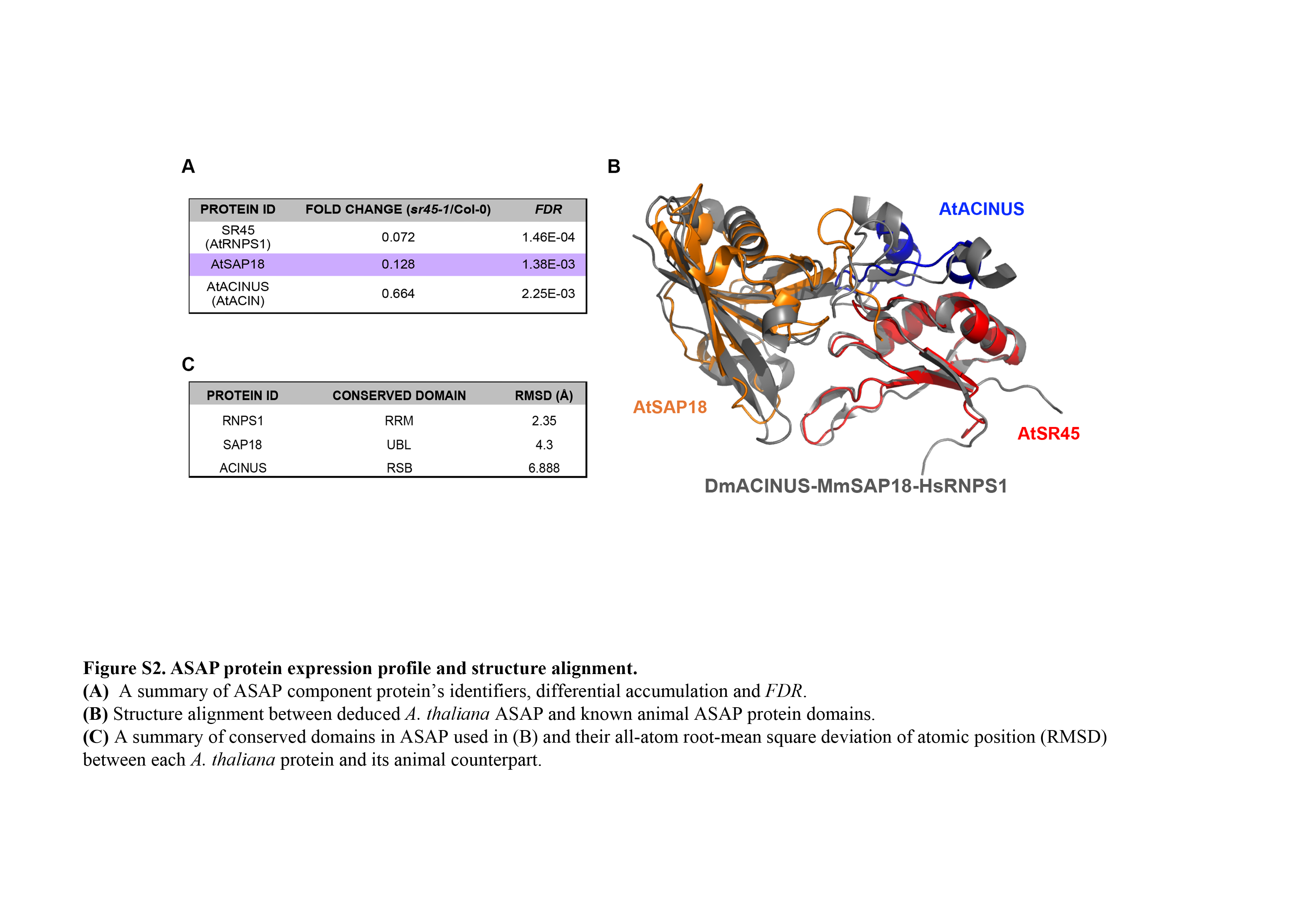

Supplement: Supplementary file 2 [file Image_2.tif]

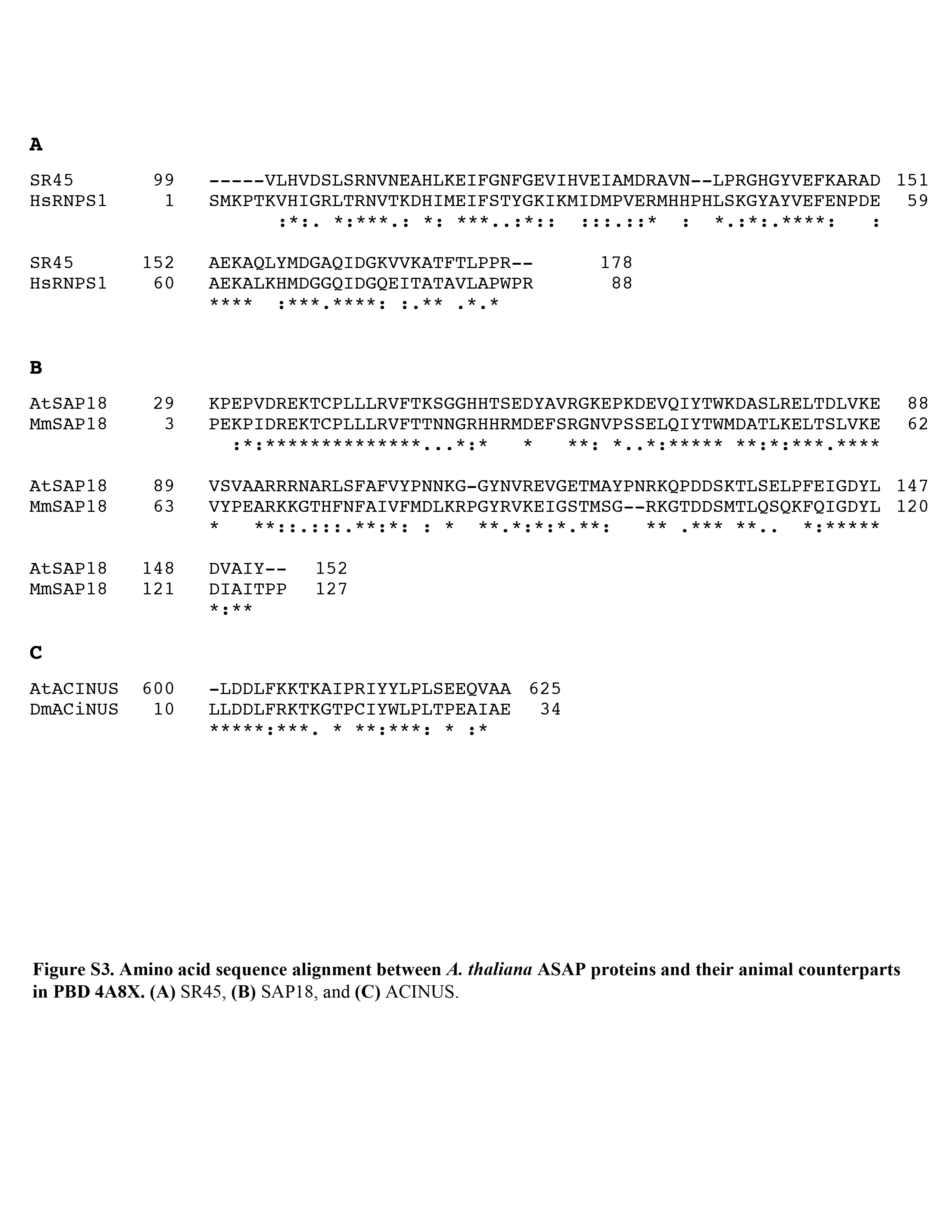

Supplement: Supplementary file 3 [file Image_3.tif]

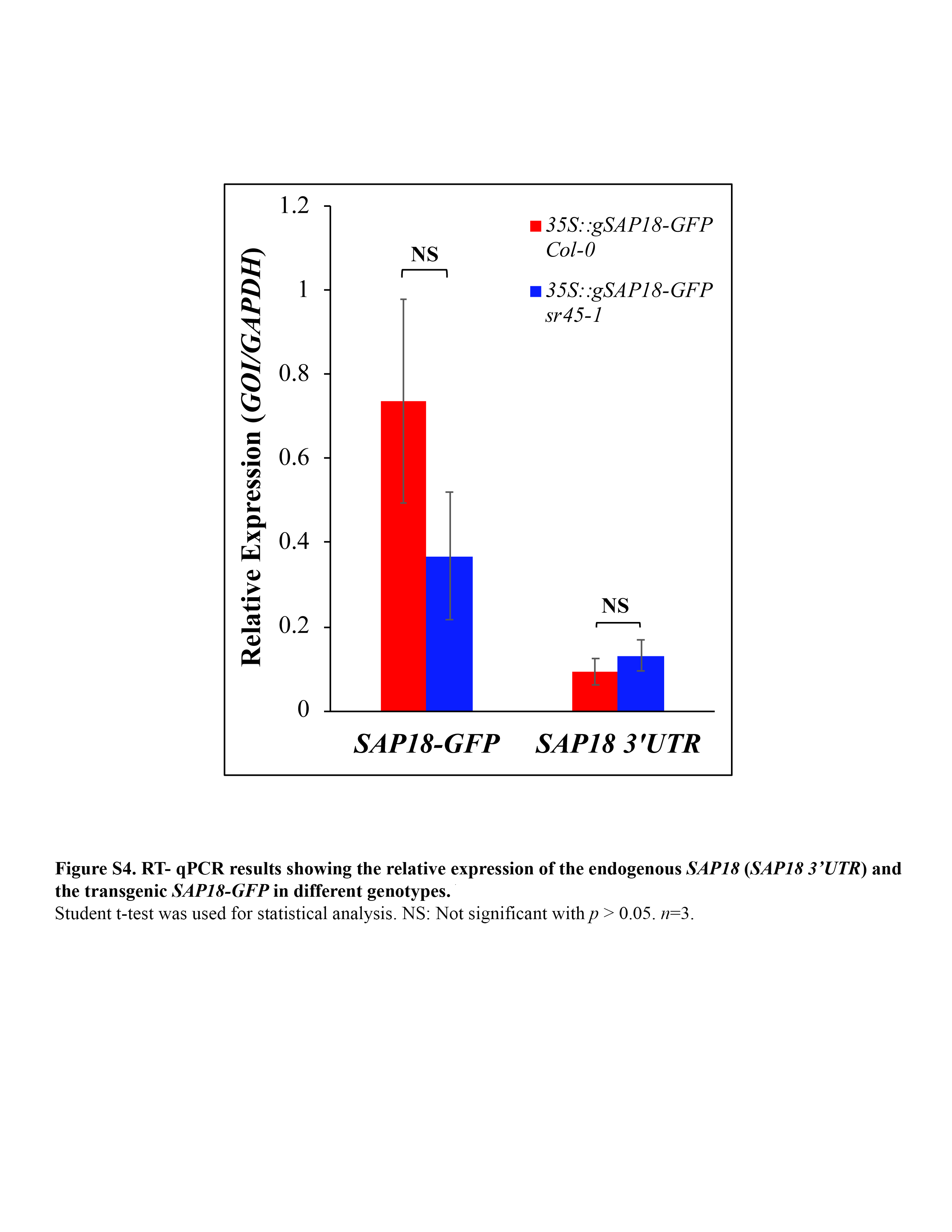

Supplement: Supplementary file 4 [file Image_4.tif]
